# CCIVR facilitates comprehensive identification of cis-natural antisense transcripts with their structural characteristics and expression profiles

**DOI:** 10.1038/s41598-022-19782-5

**Published:** 2022-09-15

**Authors:** Tatsuya Ohhata, Maya Suzuki, Satoshi Sakai, Kosuke Ota, Hazuki Yokota, Chiharu Uchida, Hiroyuki Niida, Masatoshi Kitagawa

**Affiliations:** 1 Department of Molecular Biology, Hamamatsu University School of Medicine, Hamamatsu, Shizuoka 431-3192 Japan; 2 Advanced Research Facilities & Services, Preeminent Medical Photonics Education & Research Center, Hamamatsu University School of Medicine, Hamamatsu, Shizuoka 431-3192 Japan

**Keywords:** Cell biology, Computational biology and bioinformatics, Developmental biology, Genetics, Molecular biology

## Abstract

Cis-natural antisense transcripts (cis-NATs) are transcribed from the same genomic locus as their partner gene but from the opposite DNA strand and overlap with the partner gene transcript. Here, we developed a simple and convenient program termed CCIVR (*c*omprehensive *c*is-NATs *i*dentifier *v*ia *R*NA-seq data) that comprehensively identifies all kinds of cis-NATs based on genome annotation with expression data obtained from RNA-seq. Using CCIVR with genome databases, we demonstrated total cis-NAT pairs from 11 model organisms. CCIVR analysis with RNA-seq data from parthenogenetic and androgenetic embryonic stem cells identified well-known imprinted cis-NAT pair, *KCNQ1*/*KCNQ1OT1*, ensuring the availability of CCIVR. Finally, CCIVR identified cis-NAT pairs that demonstrate inversely correlated expression upon TGFβ stimulation including cis-NATs that functionally repress their partner genes by introducing epigenetic alteration in the promoters of partner genes. Thus, CCIVR facilitates the investigation of structural characteristics and functions of cis-NATs in numerous processes in various species.

## Introduction

Natural antisense transcripts (NATs), first discovered in bacteria as early as 1981^1^, are the transcripts encoding complementary sequences to other RNA transcripts^2,3^. In contrast to trans-NATs whose partner genes are transcribed from different genomic loci, cis-NATs fully or partially overlap their partner genes but are transcribed from the opposite DNA strand, and some of them function in the regulation of gene expression^4,5^. Cis-NATs regulate gene expression at different levels. At the level of transcriptional regulation, a cis-NAT negatively regulates its partner gene by interfering with recruitment of RNA polymerase II to its overlapping region (e.g., *Airn*^6^ and *qrf*^7^), by depositing repressive epigenetic modifications on the promoter of its partner gene (e.g., *Tsix*^8,9^), and by recruiting epigenetic repressors, such as G9a, PRC2, and PRC1 (e.g., G9a and PRC2 by *Kcnq1ot1*^10^; PRC2 by *ANRIL*^11^; PRC1 by *ANRIL*^12^). In contrast, cis-NATs positively regulate their partner genes by forming RNA-DNA-DNA triplexes that recruit active epigenetic regulators to the regulatory elements of the partner gene (e.g., *KHPS1*^13^ and *TCF21*^14^). At the level of post-transcriptional regulation, cis-NATs positively regulate their partner genes by forming an RNA duplex, which stabilizes its partner gene to mask RNase and miRNA degradation (e.g., *BACE-AS1*^15^ and *Sirt1 AS*^16^). At the level of translation, cis-NATs negatively regulate their partner genes by masking ribosomal pairing (e.g., *MAPT-AS1* and MIR_NATs^17^). In contrast, cis-NATs positively regulate their partner genes by forming duplex RNAs that recruit the partner transcript to heavier polysomes (e.g., *AS Uchl1*^18^ and SINEUPs^18,19^).

Cis-NATs can be divided into four types according to their structural characteristics. In the “embedded type”, the entire transcription unit of the antisense gene is embedded in the transcription unit of the sense gene. In contrast, in the “fully-overlapped type”, the transcription unit of the antisense gene covers the entire sense gene. In the “head-to-head type”, sense and antisense genes partially overlap only at their 5′ ends, while in the “tail-to-tail type”, the partial overlap is only at their 3′ ends. To investigate the function of cis-NATs, it is important to determine their structural characteristics, including the distance between the promoters of the sense and antisense genes and whether the antisense transcription unit contains a regulatory element of the sense gene, such as its promoter, enhancer, miRNA targeting sequence, or ribosome binding sites.

Therefore, to elucidate the function of cis-NATs, it is critical to investigate both structural characteristics and expression profiles of cis-NAT pairs simultaneously. RNA-seq has become a common technique to investigate genome-wide gene expression, and genome-wide sequencing data is accumulating in databases, such as those curated by ENCODE^20^ and FANTOM^21^. To date, whole genomes and transcriptomes from more than 2,000 species, including subspecies and strains, are deposited in Ensembl^22^ and NCBI. The transcriptome data include the locational information of each gene, including chromosome location, strand direction, transcription start sites (TSS), and transcription termination sites (TTS). Using the locational information of each gene in transcriptome data, it is theoretically possible to simultaneously investigate expression profiles and structural characteristics of cis-NATs. Identification of comprehensive cis-NATs with their original pipelines has been reported from multiple species including Arabidopsis^23^, rice^24^, maize^25^, and sugarcane^26^, as well as three kinds of mammals such as human, mouse, and rat^27^, and 10 different species^28^; however, the source codes for the computational program are not available for researchers. In contrast, the source code of some of the bioinformatics tools is available: NASTI-seq^29^, written in R, allows reliable detection of cis-NATs using variable error rate of the strand-specific protocol; NATpipe^30^, written in Perl, allows systematical discovery of NATs from de novo assembled transcriptomes; BEDTools^31^, written in C +  + , allows identification of overlapped cis-NAT pairs based on genome annotation. While these tools offer a reliable method for such analysis, they are not amenable to identifying the cis-NATs with their expression profiles and structural characteristics including “embedded”, “fully-overlapped”, “head-to-head”, and “tail-to-tail”.

Here, we developed a simple and convenient program termed CCIVR (*c*omprehensive *c*is-NATs *i*dentifier *v*ia *R*NA-seq data) that enables the identification of total cis-NAT information with their structural characteristics based on its locational information with or without expression profiling obtained from processed RNA-seq data. CCIVR provides a novel tool to simultaneously investigate the function and structural characteristics of cis-NATs in numerous processes in various species.

## Results

### Overview of CCIVR and its principles of operation

**Figure 1:**
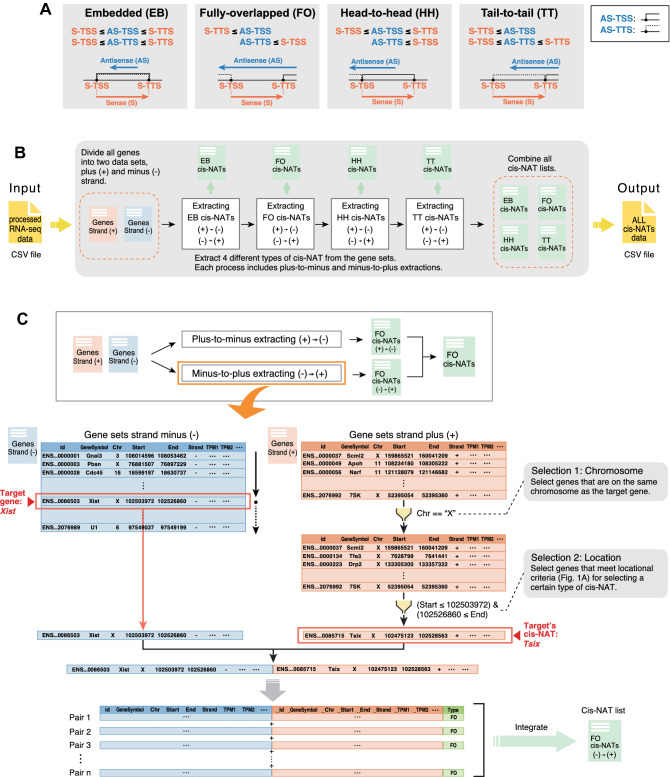
Overview of CCIVR and the processing steps of its pipeline. (**A**) Definition of four types of cis-NAT analyzed in this study, “embedded”, “fully-overlapped”, “head-to-head”, and “tail-to-tail”, which can be identified by CCIVR. TSS: transcription start site; TTS: transcription termination site. (**B**) Overview of CCIVR. The program generates a list of all cis-NAT types from a processed RNA-seq data. The processed RNA-seq data must contain five different gene annotation columns including “id”, “Chr”, “Strand”, “Start”, and “End”. Attaching information for expression profiling obtained from RNA-seq analysis is also available such as “TPM”, “FPKM”, “fold-change”, and “padj”. For details, please see README.md file placed at https://github.com/CCIVR/ccivr. (**C**) An example of the process for a target gene, *Xist*, and identification of its cis-NAT, *Tsix*, during the process that identifies fully-overlapped cis-NATs from minus to plus strand.

To simultaneously investigate genome-wide structural characteristics and expression profiling of cis-NAT pairs, we developed CCIVR. Four types of cis-NAT, embedded (EB), fully-overlapped (FO), head-to-head (HH), and tail-to-tail (TT) are defined in CCIVR according to the criteria shown in Fig. 1A. Previous studies did not separate EB and FO cis-NATs because they defined “paired gene sets” as cis-NATs^3,4^. Here, we defined “antisense transcripts” as cis-NATs and this is why we separated the types as described above. Furthermore, in this study, the criteria of identified cis-NATs were based on their structural characteristics only, and were not related to their RNA type, such as protein coding, long non-coding RNA (lncRNA), miRNA, and pseudogene.

The CCIVR process runs in a step-by-step manner (Fig. 1B). The input file contains every gene’s locational information, including chromosome location, strand direction, TSS, and TTS, obtained from the Ensembl database, as well as expression profiles, such as FPKM (fragments per kilobase of exon per million mapped reads) or TPM (transcripts per kilobase million), obtained from processed RNA-seq data using a peer-reviewed tool such as RSEM^32^ (for details, please see README.md file placed at https://github.com/CCIVR/ccivr). The input file is first divided into two groups of data sets according to whether the genes are on the plus ( +) or minus (-) strand. Subsequently, the four cis-NAT types, EB, FO, HH, and TT, are sequentially extracted from-plus-to-minus and from-minus-to-plus strands to generate transient data that list each type of cis-NAT. Finally, all of the cis-NATs are combined to generate an output file that contains all cis-NAT data.

As an example from eight CCIVR processes, extraction of FO cis-NATs from-minus-to-plus strand is shown in Fig. 1C. A mouse dataset that contains 55,146 genes (Ensembl GRCm39) was subjected to the process. The *Xist* gene was chosen as an example target gene on the minus strand, and the *Tsix* gene was chosen as an example of an identified FO cis-NAT on the plus strand. Since the *Xist* gene is on the X-chromosome, only the genes on the minus strand of the same chromosome were selected (selection 1: chromosome, Fig. 1C). The selected genes on the minus strand (1,352 genes) were subjected to the next screening that matched the criteria for FO screening of the *Xist* gene [condition: (AS-TSS ≤ 102,503,972) & (102,526,860 ≤ AS-TTS)] (selection 2: location, Fig. 1C). *Xist* and *Tsix* information was combined as paired data with their structural relationship information as “FO”, and all of the identified cis-NAT pairs were integrated as an FO cis-NAT list. With mouse datasets, a total of 317.4 million gene-to-gene comparisons were performed to accomplish the CCIVR analysis.

### A comprehensive study of cis-NATs in model organisms

**Figure 2:**
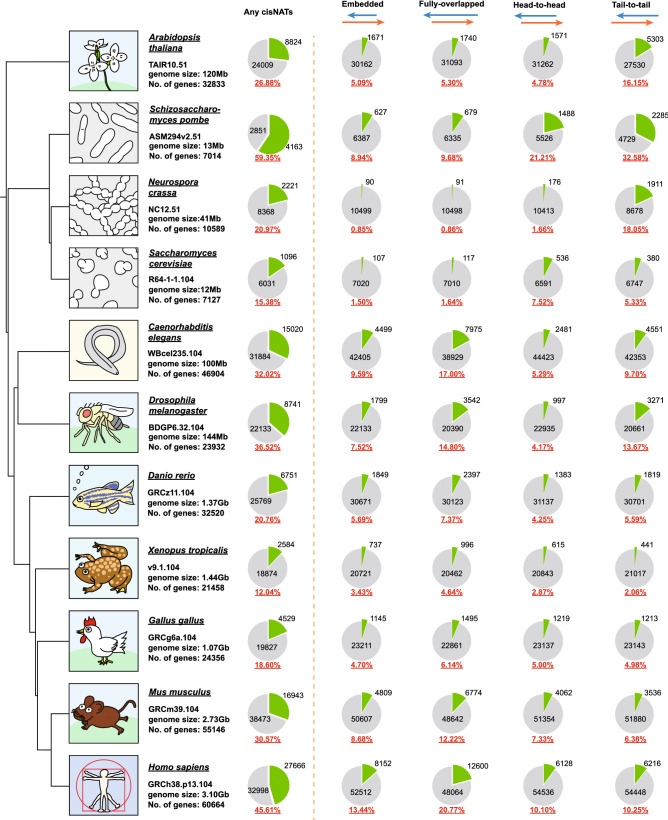
CCIVR enables identification of total cis-NATs: demonstration with representative model organisms. The percentage and number of genes that possess each type of cis-NAT (any cis-NAT, embedded, fully-overlapped, head-to-head, or tail-to-tail) from eleven different model organisms are shown. Red and blue arrows represent sense transcripts and their antisense transcripts, respectively. The genome data version from Ensembl/Ensembl_plant/Ensembl_fungi is shown below the name of each species. The phylogenetic tree was generated by phyloT_v2 (https://phylot.biobyte.de).

Subsequently, we attempted total cis-NAT identification from multiple model organisms with CCIVR. Ensembl (release 105), Ensembl plant (release 52), and Ensembl fungi (release 52), contain data for 311, 119, and 1,506 species (including sub-species and strains), respectively. From these, we chose 11 genetically well-studied representative model organisms for CCIVR analysis (Fig. 2, Supplementary Dataset File 1). We found that the percentage of cis-NAT-containing genes tended to increase from lower to higher organism complexity among each of fungi (*S. cerevisiae*, *N. crassa*, and *S. pombe*), invertebrate (*C. elagans* and *D. Melanogaster*), and vertebrate species (*D. rerio*, *X. tropicalis*, *G. gallus*, *M. musculus*, and *H. sapiens*) (Fig. 2: please note that this was not the case for *D. rerio*). Interestingly, there tended to be an inverse correlation with the percentage of protein-coding genes (Fig. S1), indicating that the existence of non-coding RNA is a reason to increase the percentage of cis-NATs. However, CCIVR analysis with only protein-coding genes also showed this tendency (Fig. S2, Supplementary Dataset File 2), indicating that lncRNA is not the only reason for the positive correlation between the percentage of cis-NATs and evolutionary complexity. Although the completeness of these databases might vary among species, which reflects the number of cis-NATs identified, these results indicate that the percentage of cis-NATs and evolutionary complexity might be somehow correlated. Intriguingly, the positive correlation between the percentage of cis-NATs and evolutionary complexity could be also confirmed by the data from a previous study that attempted to identify total cis-NATs from different species^28^; among invertebrates, the percentage of cis-NATs was 6.8% and 22.8% in worm and fly respectively, and among vertebrates, the percentage of cis-NATs was 5.2%, 6.7%, 9.7%, 28.6%, and 36.2% in zebrafish, frog, chicken, mouse, and human, respectively. The percentage of the cis-NATs tended to increase in all species in our study compared to the previous one. It may reflect the improvement of gene annotation and genome information of the moment. In summary, CCIVR facilitates the comprehensive identification of all types of cis-NAT pairs from numerous different species.

### Identification of cis-NATs that demonstrate parental-biased expression in embryonic stem cells

**Figure 3:**
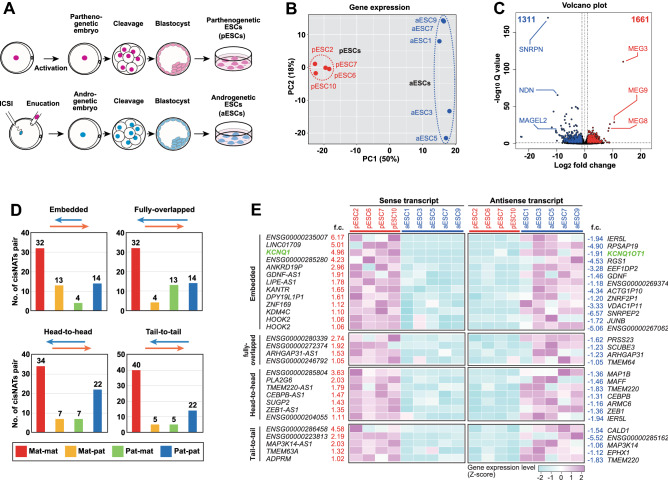
Example of CCIVR analysis I: identification of cis-NATs that demonstrate parentally-biased expression in ESCs. (**A**) Schematic representation of the procedure for establishing parthenogenetic ESCs (pESCs^34^) and androgenetic ESCs (aESCs^35^). (**B**) Principle component analysis of gene expression levels in pESCs and aESCs. The percentage of explained variation is indicated in parentheses. (**C**) Volcano plot of RNA-seq data displaying the gene expression values for pESCs compared with aESCs. Highly differentially expressed genes (DEGs) with statistical significance (padj < 0.05) in pESCs (fold change ≥ 2) and aESCs (fold change ≤ 0.5) are highlighted in red and blue, respectively. The total number of DEGs is shown. Selected known imprinted genes^35^ are also indicated. (**D**) CCIVR analysis with DEGs. The number of four kinds of cis-NAT pairs (embedded, fully-overlapped, head-to-head, tail-to-tail) with different expression patterns: maternally-highly expressed gene (mat) paired with mat (Mat-mat), mat paired with paternally-highly expressed gene (pat) (Mat-pat), pat paired with mat (Pat-mat), and pat paired with pat (Pat-pat). The numbers of each cis-NAT pair are also indicated. (**E**) Heatmap analysis of four kinds of cis-NAT pairs with inverse-correlated gene expression (Mat-pat). *KCNQ1*/*KCNQ1OT1*, highlighted in green, is a known cis-NAT pair showing imprinted expression in an inverse-correlated fashion. f.c.: fold change (log2).

A well-known process that cis-NATs are involved in is genomic imprinting, which is an epigenetic phenomenon whereby identical alleles of genes are expressed in a parent-of-origin-dependent manner^33^. We attempted to identify cis-NAT pairs that demonstrate parentally biased expression to evaluate whether the CCIVR program can identify known and/or novel imprinted cis-NAT pairs. To this end, we used published RNA-seq data from human parthenogenetic embryonic stem cells (pESCs^34^) and androgenetic ESCs (aESCs^35^), which possess only maternally or paternally inherited gene sets, respectively (Fig. 3A). (Please note that strand-specific RNA-seq data is preferable for CCIVR analysis because the origin of sequence reads from an overlap region becomes apparent. Nevertheless, non-strand-specific RNA-seq samples are applicable; indeed, this pESC and aESC RNA-seq data is non-strand-specific.) The RNA-seq samples were verified by principal component analysis (PCA) (Fig. 3B; each pESC or aESC sample was spatially gathered) and volcano plot analysis (Fig. 3C; well-known maternally and paternally imprinted genes could be identified). We defined differentially expressed genes (DEGs) as those whose difference in expression between pESCs and aESCs was statistically significant (padj < 0.05) and that the difference was more than twofold; 1,661 and 1,311 DEGs were identified in pESCs and aESCs, respectively (Fig. 3C). The processed RNA-seq data were then subjected to CCIVR analysis (Supplementary Dataset File 3), and the numbers of each type of cis-NAT pair for genes that were differentially expressed in pESCs (maternal expression or “mat”) or aESCs (paternal expression or “pat”) were counted (Fig. 3D). Then, we counted the number of all types of cis-NAT pairs that show positive or negative correlation, and 164 and 29 cis-NAT pairs were found, respectively. (please note that some of the cis-NAT pairs were redundantly represented in Fig. 3D. For example, the cis-NAT pairs showing embedded type with mat-pat expression were the same as the cis-NAT pairs showing fully-overlapped type with pat-mat expression) For all types of cis-NAT pair, the correlations with overlapping genes tended to be positive (mat-mat or pat-pat) rather than negative (mat-pat or pat-mat), consistent with previous studies^27,36^. Although positive correlation is interesting because it might indicate positive regulation of gene expression in the imprinted gene clusters, we rather focused on the negative correlation because some cis-NATs have been reported to act as negative regulators of partner genes in the imprinted gene clusters^6,10^. We analyzed all 29 cis-NAT pairs that showed negative correlation from all types of cis-NATs by heatmap analysis (Fig. 3E). Notably, among the embedded type of cis-NAT pairs, we identified the well-known functional cis-NAT, *KCNQ1OT1*, and its partner gene, *KCNQ1*, which tended to be expressed from paternal and maternal alleles, respectively (Fig. 3E), consistent with their previously reported expression patterns^37^. Taken together, we conclude that CCIVR is an effective program for identifying functional cis-NAT pair candidates from numerous deposited RNA-seq datasets.

### Identification of cis-NATs upon TGFβ stimulation

**Figure 4:**
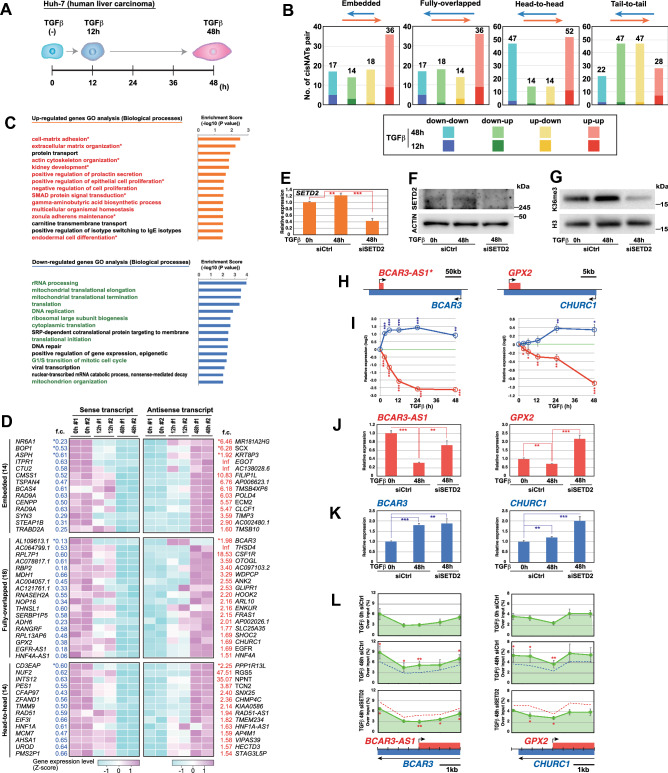
Example of CCIVR analysis II: identification of cis-NATs with TGFβ stimulation. (**A**) Schematic representation for TGFβ stimulation of Huh-7 cells. For details, see also Figure S3. (**B**) CCIVR analysis with genes that demonstrate differential expression with TGFβ stimulation [upgenes: fold change ≥ 1.5, padj < 0.05; downgenes: fold change ≤ 0.67, padj < 0.05]. The numbers of four kinds of cis-NAT pairs with the different combinations of expression pattern including down-down, down-up, up-down, and up-up (sense-antisense) are shown. (**C**) GO analysis of biological processes with genes from differentially expressed cis-NAT pairs. The top 15 biological processes are shown. Processes related to TGFβ signaling in up-regulated genes are in red and, among them, those related to EMT are indicated with an asterisk (*). Processes related to cell growth in down-regulated genes are in green. (**D**) Heatmap analysis of cis-NAT pairs with inverse-correlated gene expression (down-up). Three kinds of cis-NATs, embedded, fully-overlapped, and head-to-head are shown. Fold change (f.c.) with an asterisk (*) indicates significant differential expression after TGFβ stimulation for 12 h and the other values are for 48 h after TGFβ stimulation. (**E**–**G**) Confirmation of SETD2 expression by RT-qPCR (**E**) and western blotting (**F**) and the level of H3K36me3 by western blotting (**G**) in SETD2 knockdown samples. Images for western blotting were cropped for improving the clarity and conciseness. Original blots are presented in Supplementary Figure S5. (**H**) Maps of cis-NAT pairs, *BCAR3-AS1*/*BCAR3* and *GPX2*/*CHURC1*. *: *BCAR3-AS1* replaced *AL109613.1* by revisiting the latest version of Ensembl, Human GRCh38p13. (**I**) Expression dynamics of *BCAR3-AS1*/*BCAR3* and *GPX2*/*CHURC1* cis-NAT pairs upon TGFβ stimulation for 48 h. (**J, K**) RT-qPCR analysis for sense transcripts (**J**) and their cis-NATs (**K**) upon TGFβ stimulation with or without SETD2 knockdown. (**L**) ChIP-qPCR analysis of H3K36me3 accumulation at the promoter of sense transcripts. For comparison, blue and red dashed lines representing the level of H3K36me3 accumulation in “TGFβ 0 h siCtrl” and “TGFβ 48 h siCtrl” are shown. (**E**, **I**–**L**) Means ± SD across biological replicates are shown (**E**, **I**–**K**: n = 3; L: n = 5). *p < 0.05, **p < 0.01, ***p < 0.001, comparisons as indicated (**E**, **I**–**K**) and by dashed lines (**L**); Student’s t-test. Normalized to *GAPDH* (**E**, **J**–**K**).

To further evaluate CCIVR-mediated identification of cis-NATs involved in a biological process, we chose to examine the TGFβ signaling pathway, which induces epithelial-mesenchymal transition (EMT) and apoptosis^38^. To this end, we performed RNA-seq of the human hepatocellular carcinoma cell line, Huh-7, with or without TGFβ stimulation for 12 h and 48 h (Fig. 4A and S3A), with EMT confirmed by morphological examination (Fig. S3B and C). These RNA-seq samples were prepared as “strand-specific” to improve the accuracy of mapping at the overlap region. Reproducibility between duplicated samples was confirmed by PCA analysis (Fig. S3D) and DEGs were identified by volcano plot analysis (Fig. S3E and F). We defined DEGs as those whose difference in expression between sample groups was statistically significant (padj < 0.05) and that the difference was more than 1.5-fold for up-regulated genes and less than 0.67-fold for down-regulated genes. Then, following GO analysis (Fig. S3G) and confirmation of epithelial/mesenchymal marker gene expression (Fig. S3H), which indicates proper TGFβ responses, the processed RNA-seq data were subjected to CCIVR analysis (Supplementary Dataset File 4) and the numbers of each type of DEG constituting cis-NAT pairs were counted (Fig. 4B). These genes were subjected to GO analysis, and we found that TGFβ signaling-related genes, including genes involved in EMT, were enriched in up-regulated genes and that cell growth-related genes were enriched in down-regulated genes (Fig. 4C). This indicated that cis-NAT genes that are differentially expressed by TGFβ stimulation are involved in TGFβ-related biological processes.

We subsequently attempted to discover novel cis-NATs that regulate the expression of their partner genes. The murine *Tsix* gene is a cis-NAT that coordinates the initiation of X chromosome inactivation by negatively regulating its partner gene, *Xist*^39–41^. We have studied the mechanism of *Tsix* action and found that a histone modification, H3K36me3, accompanied by *Tsix* transcription is required for *Xist* repression^8,9^. We therefore focused on the *Tsix*-like regulation system from among the multiple kinds of regulation system that cis-NATs possess. We selected cis-NAT pairs whose expression was negatively correlated as down-up [the expression of sense transcript is down-regulated whereas its antisense transcript (cis-NAT) is up-regulated upon TGFβ stimulation], and they were subjected to heatmap analysis (Fig. 4D; please note that the tail-to-tail group was omitted owing to limited space). Given that *Tsix* transcription running through the *Xist* promoter is required for its function^42^, we focused on only fully-overlapped and head-to-head cis-NATs because their transcription runs through the promoters of their partner genes. These genes were subjected to further analysis to investigate whether the down-regulation observed in the sense genes was dependent on SETD2 histone methyltransferase, which catalyzes H3K36me3 modification (Fig. S4: please note that *BCAR3-AS1* and *AC046134.2* replaced *AL109613.1* and *AC097103.2*, respectively, after revisiting the latest version of Ensembl, Human GRCh38.p13). The efficiency of SETD2 knockdown was confirmed by decreased levels of its mRNA (Fig. 4E) and protein (Fig. 4F), and reduced catalysis of H3K36me3 modification (Fig. 4G). Based on the expression of 32 cis-NAT pairs, we chose nine cis-NAT pairs (*BCAR3-AS1*/*BCAR3*, *RBP2*/*AC097103.2*, *THNSL1*/*ENKUR*, *SERBP1P5*/*FRAS1*, *ADH6*/*AP002026.1*, *GPX2*/*CHURC1*, *EGFR-AS1*/*EGFR*, *CFAP97*/*SNX25*, and *UROD*/*HECTD3*), and investigated their dynamic expression upon TGFβ stimulation and then after SETD2 depletion. We also assessed alteration of H3K36me3 accumulation in the promoters of sense genes following SETD2 depletion. We identified two cis-NAT pairs, *BCAR3-AS1*/*BCAR3* and *GPX2*/*CHURC1*, that demonstrated statistically significant alterations in these experiments (Fig. 4H–L). Interestingly, both of these cis-NAT types were “fully-overlapped”, which is the same as *Tsix*. Furthermore, their structures resembled *Tsix/Xist*^40^ in that the TSS of its sense transcript is at the 3′ end of its cis-NAT (Fig. 4H). Dynamics analysis revealed that antisense and sense transcription was symmetrically altered upon TGFβ stimulation (Fig. 4I). While expression of the sense genes was significantly decreased (Fig. 4J) and that of the cis-NATs was significantly increased (Fig. 4K) upon TGFβ stimulation, H3K36me3 modification was significantly increased at the promoter regions of sense genes (Fig. 4L). When SETD2 was depleted, the accumulated H3K36me3 was significantly reduced (Fig. 4L) accompanied by derepression of the sense genes (Fig. 4J). Importantly, the derepression was not because of the decrease in their cis-NAT expression (Fig. 4K). Taken together, these results indicate that the transcription of cis-NATs, *BCAR3* and *CHURC1*, negatively regulate their partner genes, *BCAR3_AS1* and *GPX2*, by promoting H3K36me3 modification within the promoters of each partner gene.

BCAR3 is involved in anti-estrogen resistance in breast cancer cells^43^. Although stable overexpression of BCAR3 does not lead to a typical EMT phenotype, it results in down-regulation of cadherin-mediated adhesion and augmentation of fibronectin expression^44^, suggesting that it positively regulates part of the EMT phenotype. In contrast, the function of *BCAR3-AS1* is obscure. It would be interesting to elucidate whether BCAR3 promotion of the EMT phenotype is through repression of *BCAR3-AS1*. CHURC1 is a zinc finger transcriptional activator^45^. Its cis-NAT, *GPX2*, encodes a glutathione peroxidase (GPX) that possesses glutathione-dependent hydrogen peroxidase reducing activity^46^. GPX2 is known as a negative regulator of apoptosis^47^; therefore, investigation of its involvement in the progression of apoptosis by TGFβ stimulation is warranted. In summary, we used CCIVR to identify novel cis-NATs that regulate the expression of their partner genes. CCIVR analysis can therefore be used to screen and identify cis-NATs that possess specific mechanisms of action among multiple kinds of gene regulation.

## Discussion

In this study, we demonstrated that CCIVR can contribute to the identification of cis-NATs involved in the regulation of transcription. In contrast to transcription, some cis-NATs are involved in regulation at the level of translation^17–19^. Although CCIVR uses transcriptome data, such as from RNA-seq, it can also be applied to proteome data such as from quantitative mass spectrometry^48^. Therefore, CCIVR enables functional studies of cis-NATs in both transcriptional and translational regulation. Some antisense RNAs are transcribed from sequence that is upstream of the promoter of its partner gene (in a strict sense, these genes are not cis-NAT pairs because they do not overlap), and some antisense transcripts may have a role in regulating expression of their partner genes through modulating the action of their enhancers. The CCIVR program is open-source and can be readily customized for specific purposes; therefore, identifying such antisense RNAs is also practicable.

Many cis-NATs involved in human diseases have been reported^49,50^, and some of them are therapeutic targets. Therefore, CCIVR can contribute biomedically by identifying novel cis-NATs involved in human diseases. Compared to previous studies attempting comprehensive identification of cis-NATs using *Arabidopsis* genome data^23^, human microarray data^27^, and EST data from 10 different species^28^, our study has two advances: firstly, we updated the results by utilizing the latest genome datasets and, secondly, CCIVR is a simple, convenient, and open-source program that allows investigation of all RNA-seq and genome datasets from more than 2,000 species deposited in the NCBI and Ensembl databases. For predicting the composition of various cis-NATs by CCIVR, it depends on the accuracy of gene annotation including their structure and strand direction deposited in the databases. Furthermore, for performing the expression profile analysis of cis-NATs by CCIVR, it uses processed RNA-seq data using third-party programs such as STAR^51^ for mapping, RSEM^32^ for expression profiling, and DESeq2^52^ for statistical analysis; i.e., CCIVR scripts do not cover the full pipeline of CCIVR analysis. They are the limitations of CCIVR analysis at the moment and further improvements are required in the future.

Here, we introduced an original program termed CCIVR that simultaneously analyzes the structure of cis-NAT pairs and their expression profiles. We believe that CCIVR will drive the study of cis-NATs to elucidate their mechanisms of action and functions in numerous processes in various species.

## Materials and methods

### CCIVR analysis of model organisms

All of the gtf files from 11 species were downloaded from Ensembl plant (https://plants.ensembl.org/index.html; *Arabidopsis thaliana*: TAIR10, release 51), Ensembl Fungi (https://fungi.ensembl.org/index.html; *Schizosaccharomyces pombe*: ASM294v2, release 51, *Neurospora crassa*: NC12, release 51, *Saccharomyces cerevisiae*: R64-1-1, release 104), and Ensembl (https://www.ensembl.org/index.html; *Caenorhabditis elegans*: WBcel235, release 104, *Drosophila melanogaster*: BDGP6.32, release 104, *Danio rerio*: GRCz11, release 104, *Xenopus tropicalis*: v9.1, release 104, *Gallus gallus*: GRCg6a, release 104, *Mus musculus*: GRCm39, release 104, *Homo sapiens*: GRCh38.p13, release 104). From the gtf files, only the gene information listed in the feature column was extracted and was converted to a csv file using Python (ver 3.8.8) and one of its modules, gtfparse (ver 1.2.1). To avoid the duplication of the genes to be analyzed, only ena and PomBase were used from *N. crassa* and *S. pombe*, respectively, as their gene_source. Concerning other species, every gene_source was used for the CCIVR analysis because no duplication was observed. The number of genes was counted from gene_id but not GeneSymbol because we found that gene_id was unique to every gene while this was not the case for a few genes in GeneSymbol. The phylogenetic tree was generated by phyloT-v2^53^ (https://phylot.biobyte.de).

### RNA-seq analysis of pESCs and aESCs

The SRA files used for pESC and aESC analysis were downloaded from NCBI (https://www.ncbi.nlm.nih.gov) and are listed in Supplemental Table S1. Low-quality RNA-seq reads and adaptor sequences were removed using Trim Galore! (version 0.6.7) with the default condition. Sequence reads were aligned to the human reference genome (GRCh38/hg38) using STAR^51^ (version 2.7.9a) by the default condition with an option that allows up to three mismatches (-outFilterMismatchNmax 3), as previously described^35^. For each gene, transcripts per kilobase million (TPM) was calculated by RSEM^32^ (version 1.3.3) using the “rsem-calculate-expression” command with the default condition. Differential expression analysis (Wald test), PCA plot analysis, and volcano plot analysis were performed using DESeq2^52^ with the default condition (Bioconductor version: Release 3.13). Heatmaps were generated using the R “*gplots*” function.

### RNA-seq analysis of Huh-7 cells

Total RNA was purified using an RNeasy Mini kit (Qiagen, Hilden, Germany). RNA quality was measured using NanoDrop spectrophotometry (Thermo Fisher Scientific, Waltham, MA, USA) and its quantity was measured using the TapeStation Automated Electrophoresis System (Agilent Technologies, Santa Clara, CA, USA). All RNA-seq procedures, including library construction, purification, library quality control and quantification, sequencing cluster generation, high-throughput sequencing, and result generation, which included PCA and volcano plotting, were performed by Genewiz Biotechnology Co. Ltd (https://www.genewiz.com). Gene expression levels were measured by reading density and FPKM (fragments per kilobases per million reads) was calculated based on the read counts from HT-seq (V 0.6.1).

### GO analysis

Gene ontology (GO) term enrichment analyses (biological processes) were performed using the bioinformatics tool, DAVID (ver 6.8)^54–56^.

### Cell culture and reagents

Human hepatoma cell line Huh-7 (JCRB0403) was purchased from JCRB Cell Bank (National Institute of Biomedical Innovation, Osaka, Japan), grown in Dulbecco’s modified Eagle’s medium (DMEM) (Sigma-Aldrich, St. Louis, MO, USA) supplemented with 10% fetal bovine serum (FBS) (Sigma-Aldrich) and 1 × penicillin/streptomycin (Meiji Seika Pharma Co., Ltd., Tokyo, Japan) at 37 °C under an atmosphere containing 5% CO_2_. Recombinant hTGFβ1 (240-B; R&D systems, Minneapolis, MN, USA) was added to a final concentration of 10 ng/ml for TGFβ stimulation. A BZ-8000 phase-contrast microscope (Keyence, Osaka, Japan) was used to monitor morphological changes upon TGFβ stimulation.

### RNA interference

Huh-7 cells were transfected with *SETD2* siRNA (si*SETD2*) or control siRNA (siCtrl) using Lipofectamine RNAiMAX (Invitrogen, Waltham, MA, USA), in accordance with the manufacturer's protocol. At 48 h post-transfection, the cells were again transfected with *SETD2* siRNA or control siRNA as per the first transfection. At 24 h after the second transfection, the cells were subjected to with or without TGFβ stimulation. *SETD2* siRNA and negative control siRNA (non-targeting pools) were purchased (si*SETD2*: L-012448-00-0005, siCtrl: D-001810-10-05; Horizon discovery, Cambridge, UK). The *SETD2* siRNA consisted of four different oligonucleotides with the following target sequences: 5′-UAA AGG AGG UAU AUC GAA U-3′ (J-012448-05); 5′-GAG AGG UAC UCG AUC AUA A-3′ (J-012448-06); 5′-GCU CAG AGU UAA CGU UUG A-3′ (J-012448-07); and 5′-CCA AAG AUU CAG ACA UAU A-3′ (J-012448-08). The nucleotide sequence of the control siRNA consisted of four different oligonucleotides with the following non-targeting sequences: 5′-UGG UUU ACA UGU CGA CUA A-3′; 5′-UGG UUU ACA UGU UGU GUG A-3′; 5′-UGG UUU ACA UGU UUU CUG A-3′; and 5′-UGG UUU ACA UGU UUU CCU A-3′.

### RT-qPCR

Total RNA was purified using an RNeasy Mini Kit (Qiagen). For RT-qPCR, cDNA was prepared using SuperScript II reverse transcriptase (Invitrogen) with random primers (Invitrogen). RT-qPCRs were performed in duplicate using Thunderbird SYBR qPCR mix (Toyobo, Osaka, Japan) with the primers listed in Supplemental Table S2 on a StepOnePlus Real-Time PCR system (Life Technologies, Carlsbad, CA, USA). The standard curve method was used for quantification and expression levels were normalized against *GADPH*.

### Western blot analysis

For SETD2, western blot analysis was performed as previously described^57^ with minor modification. In brief, cells were lysed with RIPA buffer in the presence of a protease inhibitor cocktail (cOmplete™; Roche, Basel, Switzerland). Lysed cells were rotated at 4 °C for 20 min and sonicated using a UCS-250 Bioruptor (Cosmobio, Tokyo, Japan). The sonication conditions were as follows: high, on 30 s/off 30 s, eight cycles. After collection of the supernatant by centrifugation the protein concentration was measured by a DC protein assay (Bio-Rad, Hercules, CA, USA). After denaturation of the cell lysate by diluting to final 1 × using 4 × SDS sample buffer [255 mM Tris–HCl (pH 6.8), 12% SDS, 40% glycerol, 20% β-mercaptoethanol, and 0.01% bromophenol blue] and incubation at 95 °C for 8 min, the cell lysate was separated by SDS-PAGE electrophoresis and transferred onto a polyvinylidene difluoride (PVDF) membrane (Immobilon-P IPVH00010; Millipore, Burlington, MA, USA), followed by immunoblotting with a primary α-SETD2 antibody (#EB08118; Everest Biotech, Oxford, UK) and a secondary α-Goat IgG, HRP Conjugate antibody (V805A; Promega, Madison, WI, USA), or an α-β-actin mAb, HRP conjugated antibody (289-99361; FUJIFILM Wako Pure Chemical Corp., Osaka, Japan). The signals were visualized using Clarity™ Western ECL Substrate (Bio-Rad) and the ChemiDoc Touch imaging system (Bio-Rad). H3K36me3 was detected as previously described^8^. In brief, cells were lysed with Triton extraction buffer (0.5% Triton X-100 and 0.02% NaN_3_ in PBS) in the presence of a protease inhibitor cocktail (cOmplete™; Roche) on ice for 10 min. After centrifugation, the pellet was washed once with Triton extraction buffer. The pellet was resuspended with 0.2 N HCl, and the histone protein was extracted by rotation at 4 °C overnight. After collection of the supernatant by centrifugation, the protein concentration was measured by a Bradford assay (Bio-Rad). After denaturation of the cell lysate by diluting to final 1 × using 4 × SDS sample buffer and incubation at 95 °C for 8 min, the cell lysate was separated by SDS-PAGE electrophoresis and transferred onto a 0.2 μm pore nitrocellulose membrane (Whatman PROTRAN; Merck, Darmstadt, Germany), followed by immunoblotting with a primary α-H3K36me3 antibody (ab9050; Abcam, Cambridge, UK) and a secondary α-Rabbit IgG (H + L), HRP Conjugate antibody (W401B, Promega) or a primary α-H3 antibody (#39,763; Active motif, Carlsbad, CA, USA) and a secondary α-Mouse IgG (H + L), HRP Conjugate antibody (W402B, Promega). The signals were visualized as described above.

### ChIP-qPCR

ChIP was performed with a commercial kit (SimpleChIP Enzymatic Chromatin IP Kit; Cell Signaling Technology, Danvers, MA, USA) in accordance with the manufacturer’s procedure. After de-crosslinking and proteinase K treatment, DNA was purified using phenol–chloroform extraction and ethanol precipitation with the co-precipitation reagent, Pellet Paint (Merck). For qPCR, see the RT-qPCR section. The primer sequences used in ChIP-qPCR assays are listed in Supplemental Table S2. The following antibody was used: α-H3K36me3 (CMA333; a gift from Dr. Naohito Nozaki, MAB Institute, Inc.).

## Supplementary Information

Supplementary Information 1.

Supplementary Information 2.

Supplementary Information 3.

Supplementary Information 4.

Supplementary Information 5.

## Data Availability

DNA sequencing data have been deposited in the DDBJ Sequence Read Archive (DRA) of the DNA Data Bank of Japan (DDBJ) with accession number DRA013542. CCIVR is available from github at https://github.com/CCIVR/ccivr.
