# Supplementary material for: CCIVR facilitates comprehensive identification of cis-natural antisense transcripts with their structural characteristics and expression profiles
Source: Sci Rep. 2022 Sep 15;12:15525. doi: 10.1038/s41598-022-19782-5 (PMC9477841; doi:10.1038/s41598-022-19782-5)
Supplement: Supplementary file 5 — Supplementary Information 5. [file 41598_2022_19782_MOESM5_ESM.pdf]

Supplementary information

CCIVR facilitates comprehensive identification of cis-natural antisense transcripts with their structural characteristics and expression profiles

Tatsuya Ohhata[1,3,*], Maya Suzuki[1,3], Satoshi Sakai[1], Kosuke Ota[1], Hazuki Yokota[1], Chiharu Uchida[2], Hiroyuki Niida[1], Masatoshi Kitagawa[1]

[1]Department of Molecular Biology, Hamamatsu University School of Medicine, Hamamatsu, Shizuoka 431-3192, Japan

[2]Advanced Research Facilities & Services, Preeminent Medical Photonics Education & Research Center, Hamamatsu University School of Medicine, Hamamatsu, Shizuoka 431-3192, Japan

[3] These authors contributed equally

*Correspondence: ohhata@hama-med.ac.jp (T.O.)

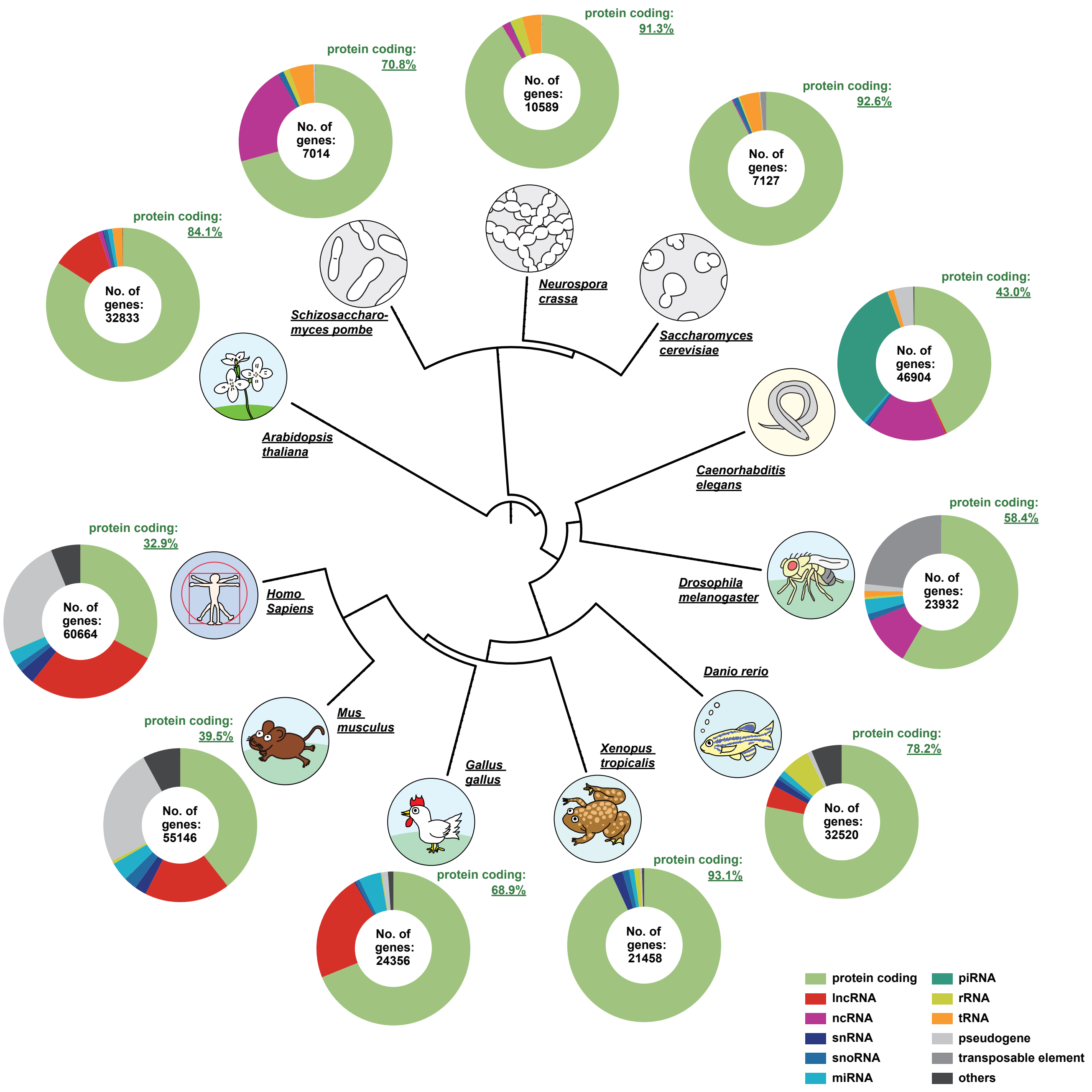

Figure S1. Types of RNA in representative model organisms

The types of RNA, as well as the percentage of protein coding genes in eleven different model organisms, are shown. The version of the genome data from Ensembl/Ensembl_plant/Ensembl_fungi is mentioned in Figure 2. The phylogenetic tree was generated by phyloT_v2 (https://phylot.biobyte.de).

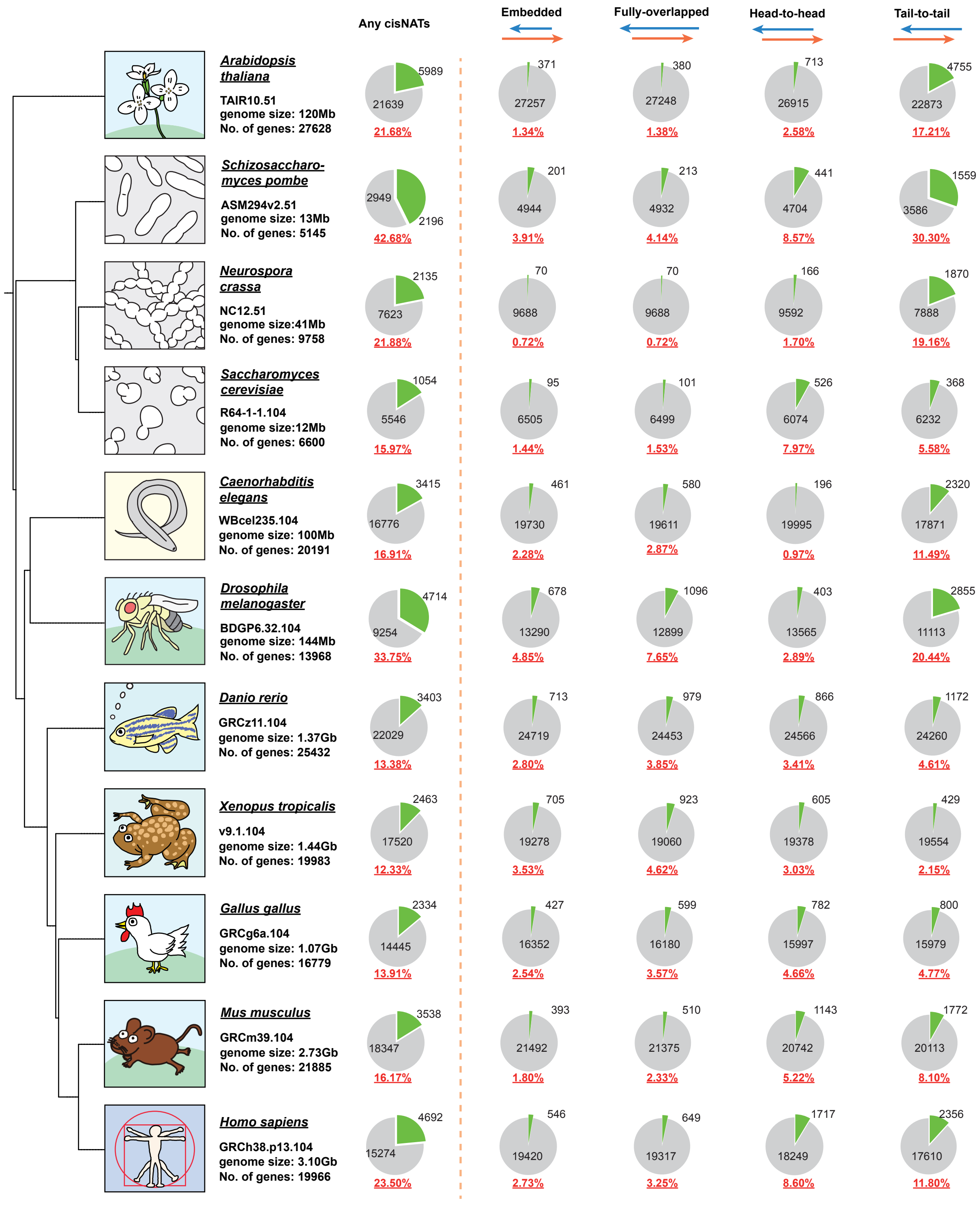

Figure S2. CCIVR analysis with representative model organisms: only protein coding genes

The percentage and number of cis-NATs (any cis-NAT, embedded, fully-overlapped, head-to-head, and tail-to-tail) from eleven different model organisms are shown as in Figure 2. Only protein coding genes were subjected to CCIVR analysis.

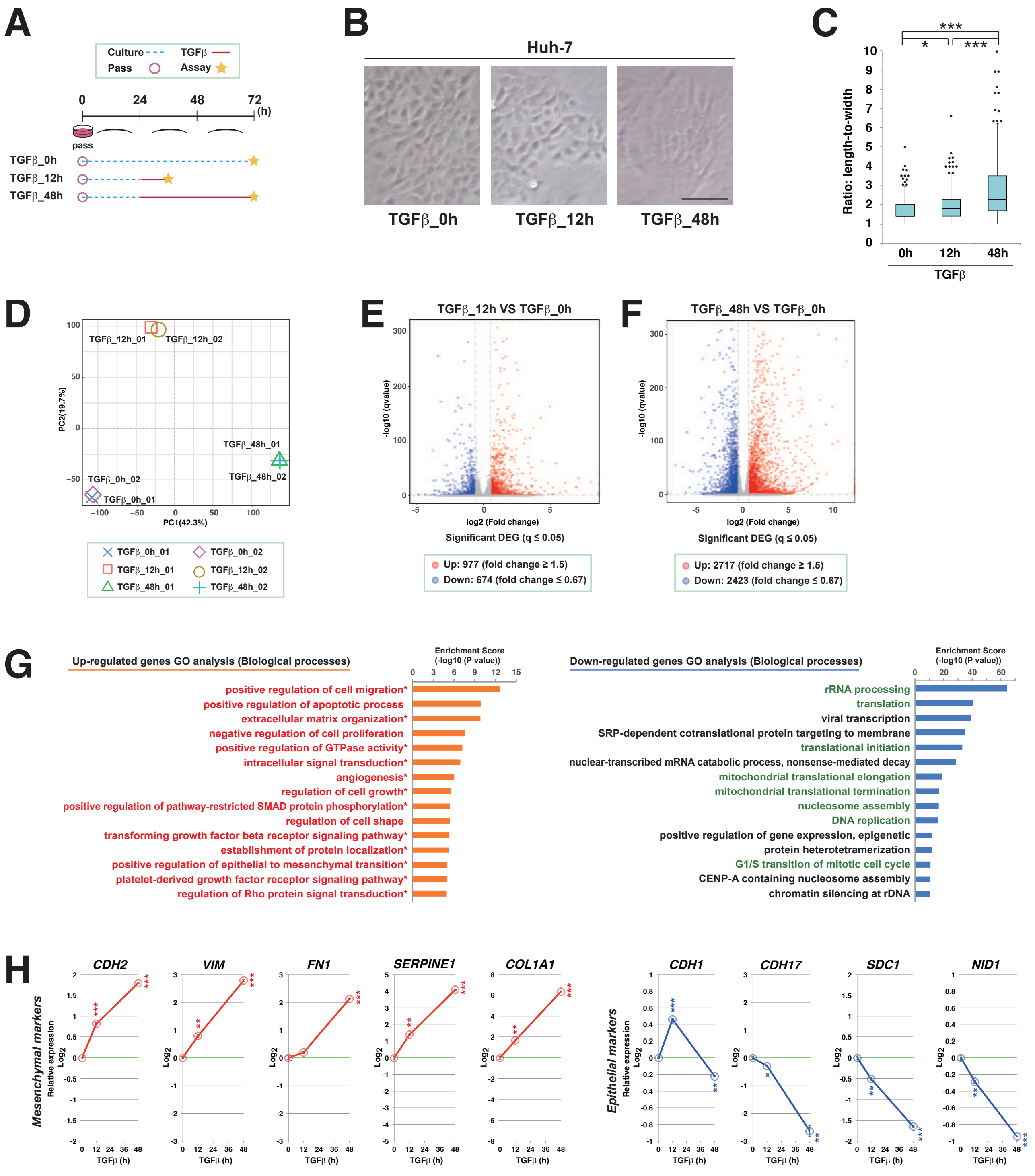

Figure S3. Identification of differentially expressed genes in EMT by RNA-seq

A Schematic representation of cell culture, the timing of TGFβ addition, and analysis.

B Representative images of Huh-7 cells stimulated with TGFβ. Scale bar: 100μm.

C Box-whisker plot or morphology changes following TGFβ stimulation. n=200, from 10 individual areas. *p < 0.05, *** < 0.001, Student’s t-test.

D Principle component analysis plot of RNA-seq. Each of the two independent samples was subjected to RNA-seq.

E,F Volcano plot of TGFβ_12h VS TGFβ_0h (E) and TGFβ_48h VS TGFb_0h (F).

G GO analysis of up-regulated and down-regulated genes by DAVID, biological processes. The top 15 biological processes are shown. Up-regulated genes related to TGFβ signaling are in red and, among them, those related to EMT are indicated by an asterisk (*). Down-regulated genes related to cell growth are in green.

H Marker expression analysis from RNA-seq (mean ± SD of two biological replicates, TGFβ_0h is set to 1). *p < 0.05, ** < 0.01, *** < 0.001, Student’s t-test.

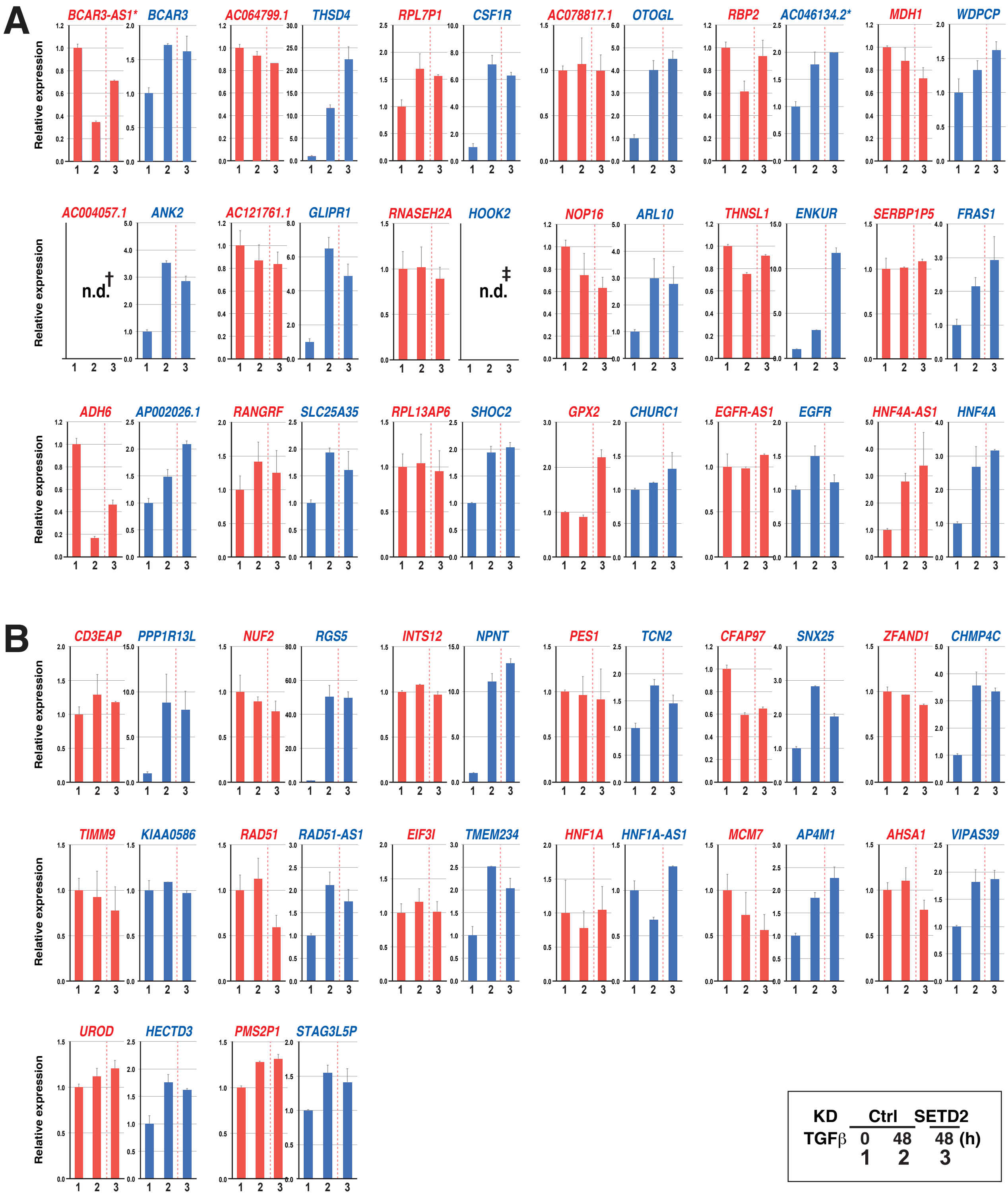

Figure S4. Expression analysis of negatively-correlated cis-NAT pairs along with SETD2 knockdown

A,B Expression analysis of genes from fully-overlapped (A) and head-to-head (B) types of cis-NAT with or without TGFβ stimulation and with SETD2 knockdown, confirmed by RT-qPCR. Normalized to *GAPDH*, two biological replicates, TGFβ_0h is set to 1. *: *BCAR3-AS1* and *AC046134.2* replaced *AL109613.1* and *AC097103.2*, respectively by revisiting the latest version of Ensembl, Human GRCh38p13. †: not determined because a specific primer set for detecting the *AC004057.1* gene could not be designed because of its repeat-rich sequences. ‡: not determined because an efficient primer set could not be obtained for the *HOOK2* gene locus for an unknown reason.

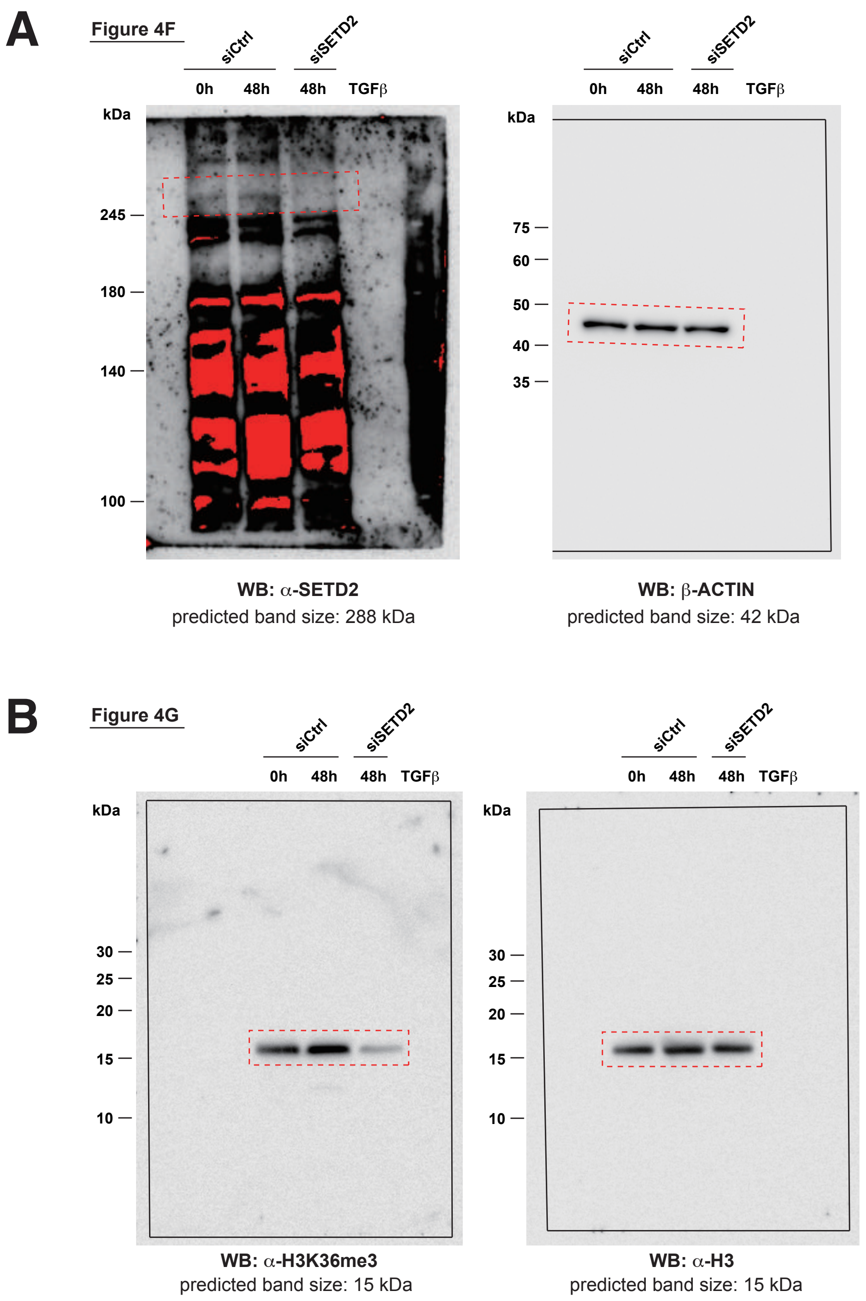

Figure S5. Uncropped images of western blotting

A,B Uncropped images of western blots from Figure 4F (A) and 4G (B), as well as the approximate cropped regions for Figure 4F and 4G indicated by dotted-red squares, are shown. The borders of the blots are surrounded by black lines since the background of the blots for β-ACTIN, H3K36me3, and H3 is faint.

Supplemental Table S1. RNA-seq data for pESCs and aESCs analysis

Sample name in this study	Original sample name	SRA/GEO accession number	Run	Number of Spots	Number of Bases	Size	References
pESC2	pES2	GSM2151895	SRR3501471	26,282,837	2.7G	1.6Gb	1
pESC6	pES6	GSM3147139	SRR7186852	32,780,769	3.3G	2.1Gb	2
pESC7	pES7	GSM2151896	SRR3501472	29,659,724	3.0G	1.9Gb	1
pESC10	pES10	GSM2151897	SRR3501473	26,528,943	2.7G	1.7Gb	1
aESC1	aES1 rep2	GSM3147104	SRR7186855	22,914,889	1.8G	651.8Mb	2
aESC3	aES3 rep2	GSM3147106	SRR7186857	34,164,901	2.5G	981.8Mb	2
aESC5	aES5 rep1	GSM3147107	SRR7186858	35,699,083	2.7G	1,008.5Mb	2
aESC7	aES7	GSM3147109	SRR7186860	26,230,351	2.0G	801.6Mb	2
aESC9	aES9	GSM3147111	SRR7186862	28,949,544	2.2G	873.3Mb	2

Supplemental Table S2. Information for primer sequences

PCR products	Primer sequence	Intron	Expected length	Application	References
SETD2		No	123 bp	RT-qPCR (Fig. 4E)	
SETD2-F1	TCAGAGGAAACCGTGAAAGC				
SETD2-R1	TGGCCTGTTTTGGAAAGTGG				
GAPDH		No	87bp	RT-qPCR (Fig. 4E, 4I-K, S4A-B)	3
GAPDH-22-F	TGCACCACCAACTGCTTAGC				
GAPDH-22-R	GGCATGGACTGTGGTCATGAG				
BCAR3-AS1		No	119bp	RT-qPCR (Fig. 4I, 4J, S4A)	
01_D_BCAR3-AS1_F1	TCCTTGGAGAACTGCAACAC				
01_D_BCAR3-AS1_R1	CAGGCTGTAGCATAACTGGTTG				
BCAR3		No	101bp	RT-qPCR (Fig. 4I, 4K, S4A)	
01_U_BCAR3_F1	CCTCTCGCGTAAATTGGAAC				
01_U_BCAR3_R1	GATGAAGCTGGGGAAACTTG				
AC064799.1		No	120bp	RT-qPCR (Fig. S4A)	
17_D_AC064799.1_F2	ACACTCGTGAAACTGCTCAG				
17_D_AC064799.1_R2	CAACTCCACCGTTGTAATGTCG				
THSD4		No	108bp	RT-qPCR (Fig. S4A)	
17_U_THSD4_F1	AAAGTGGGATGGAACCATGC				
17_U_THSD4_R1	TGGTTCCGGCAAATGCAAAC				
RPL7P1		No	87bp	RT-qPCR (Fig. S4A)	
06_D_RPL7P1_F1	AGAAGGAGGTTCCTGCTGTG				
06_D_RPL7P1_R1	ACTTCTTTCTCAGGCGCTTC				
CSF1R		No	130bp	RT-qPCR (Fig. S4A)	
06_U_CSF1R_F2	TGCAGCCCAACAACTATCAG				
06_U_CSF1R_R2	ATGACCGAAGGCAGAGTTTG				
AC078817.1		No	142bp	RT-qPCR (Fig. S4A)	
02_D_AC078817.1_F1	CGCAAAAGGCATTTCAACGC				
02_D_AC078817.1_R1	AGTGTCCTCGTACAACCTGAAC				
OTOGL		No	71bp	RT-qPCR (Fig. S4A)	
02_U_OTOGL_F2	TTGCTCAGGAAATGGCACTG				
02_U_OTOGL_R2	AATTCCACTGGCACGTACAG				
RBP2		No	75bp	RT-qPCR (Fig. S4A)	
12_D_RBP2_F1	TTGCCATCCACCACAAAC				
12_D_RBP2_R1	ATCTCCCAGGTTCCATTCTG				

PCR products	Primer sequence	Intron	Expected length	Application	References
AC046134.2		No	121bp	RT-qPCR (Fig. S4A)	
12_U__AC046134.2_F3	AATTCTCTGCAGGCCTTTGC				
12_U__AC046134.2_R3	TCCAGAAGTGTAAGCTCTCAGC				
MDH1		No	70bp	RT-qPCR (Fig. S4A)	
16_D_MDH1_F1	AGTCAGTTCCGCGGTAGAGG				
16_D_MDH1_R1	TGCGGGGACAATTTCAAC				
WDPCP		No	149bp	RT-qPCR (Fig. S4A)	
16_U_WDPCP_F1	TGTAGCACCTGAGGAAACTGTG				
16_U_WDPCP_R1	AGCAAAACTCTCGCCTCATC				
ANK2		No	70bp	RT-qPCR (Fig. S4A)	
18_U_ANK2_F1	ACCGCTCTTCACATTGCATC				
18_U_ANK2_R1	TGGCTCCTTCCTTAACAAGAAC				
AC121761.1		No	139bp	RT-qPCR (Fig. S4A)	
04_D_AC121761.1_F1	TTGCTGCTGCTCTATCTTCC				
04_D_AC121761.1_R1	AAACCCAAGCTCAGTGATCC				
GLIPR1		No	88bp	RT-qPCR (Fig. S4A)	
04_U_GLIPR1_F1	AAGCTGCACCCAAACTTCAC				
04_U_GLIPR1_R1	AGTTTGTGATGGCGGAAGAC				
RNASEH2A		No	94bp	RT-qPCR (Fig. S4A)	
11_D_RNASEH2A_F1	ACCCGCTCCTGCAGTATTAG				
11_D_RNASEH2A_R1	ACAGCGGCCTGTATTGTCTC				
NOP16		No	53bp	RT-qPCR (Fig. S4A)	
07_D_NOP16_F1	ATGGAGGTGGACATAGAGGAG				
07_D_NOP16_R1	ACATAGGGCTTCCGTACAAGC				
ARL10		No	85bp	RT-qPCR (Fig. S4A)	
07_U_ARL10_F1	ATGTGCTGGTGTTTGTGGTG				
07_U_ARL10_R1	TCCTTGTCCAGCAGCTTGTG				
THNSL1		No	121bp	RT-qPCR (Fig. S4A)	
10_D_THNSL1_F1	TTGGCTTGGGCAGAAAAGTG				
10_D_THNSL1_R1	TCGCTGTGCATGTTTATCCG				
ENKUR		No	103bp	RT-qPCR (Fig. S4A)	
10_U_ENKUR_F1	AAAAGCCTGCTGTGCCATTG				
10_U_ENKUR_R1	GCCACTCCCATGATGATATCAG				

PCR products	Primer sequence	Intron	Expected length	Application	References
SERBP1P5		No	109bp	RT-qPCR (Fig. S4A)	
13_D_SERBP1P5_F1	CGTGGCGCTTAAGAAAGAAG				
13_D_SERBP1P5_R1	ACAAGGTGGTTGCCTTTCTG				
FRAS1		No	75bp	RT-qPCR (Fig. S4A)	
13_U_FRAS1_F2	AGGCCATGTGTTTCAGGATG				
13_U_FRAS1_R2	GCAACCCCATTTCTACACAG				
ADH6		No	68 bp	RT-qPCR (Fig. S4A)	
D_005_Sense_F1	CAAGTCTGAGCTGGAGCCTC				
D_005_Sense_R1	GCCTTAGCTGCTGTCAGGAA				
AP002026.1		No	58 bp	RT-qPCR (Fig. S4A)	
D_005_Antisense_F1	TGAGAAGATTAAATAAGCCATGGGA				
D_005_Antisense_R1	ACCCTTGGAGTTTCATGTCAT				
RANGRF		No	56bp	RT-qPCR (Fig. S4A)	
03_D_RANGRF_F2	TGTGAGGGGGAAAAGAGGTTG				
03_D_RANGRF_R2	ACGCACAGGGAAGAAATAAGAG				
SLC25A35		No	113bp	RT-qPCR (Fig. S4A)	
03_U_SLC25A35_F1	AACCGAGATTGGCCAGAAAC				
03_U_SLC25A35_R1	TGGTGGATGAGAAGGTGCAC				
RPL13AP6		No	139bp	RT-qPCR (Fig. S4A)	
05_D_PRL13AP6_F2	ATGAACACCAACCCTTTCCG				
05_D_PRL13AP6_R2	TGCAGTCAAACACCTTGAGG				
SHOC2		No	90bp	RT-qPCR (Fig. S4A)	
05_U_SHOC2_F1	TACGATCAAACGGCCAAACC				
05_U_SHOC2_R1	TCTTCCCGGCATTTGTTGAG				
GPX2		No	122bp	RT-qPCR (Fig. 4I, 4J, S4A)	
08_D_GPX2_F1	AGATGTGGCCTGGAACTTTG				
08_D_GPX2_R1	CAACTTTAAGGAGGCGCTTG				
CHURC1		No	86bp	RT-qPCR (Fig. 4I, 4K, S4A)	
08_U_CHURC1_F2	TTTCAACAAGAGGCGTGCAG				
08_U_CHURC1_R2	TTTCGGTTTCTGTGGCTTCC				
EGFR-AS1		No	115bp	RT-qPCR (Fig. S4A)	
15_D_EGFR-AS1_F2	ATCTGCTGCCCTGCTAATTC				
15_D_EGFR-AS1_R2	TTTAGCTTCCTCAGCCCAAG				

PCR products	Primer sequence	Intron	Expected length	Application	References
EGFR		No	90bp	RT-qPCR (Fig. S4A)	
15_U_EGFR_F1	TAACAAGCTCACGCAGTTGG				
15_U_EGFR_R1	AAATTCCCAAGGACCACCTC				
HNF4A-AS1		No	69bp	RT-qPCR (Fig. S4A)	
09_D_HNF4A-AS1_F1	AGGCCTCCTCTGTTGCTTTG				
09_D_HNF4A-AS1_R1	TATGACCGGTGTGCAGTCAAG				
HNF4A		No	79bp	RT-qPCR (Fig. S4A)	
09_U_HNF4A_F1	AAACACTACGGTGCCTCGAG				
09_U_HNF4A_R1	AGGAGTACATGTGGTTCTTCCG				
CD3EAP		No	126bp	RT-qPCR (Fig. S4B)	
19_D_CD3EAP_F1	TGCTGCTCGGTTCTCTTGTC				
19_D_CD3EAP_R1	TCTGCAGGGGCCTGAATAAG				
PPP1R13L		No	125bp	RT-qPCR (Fig. S4B)	
19_U_PPP1R13L_F2	ACCACACCACTGGATATTCCTG				
19_U_PPP1R13L_R2	AGGCACTTAATTGGGGAGAGG				
NUF2		No	117bp	RT-qPCR (Fig. S4B)	
U_157_Antisense_F1	AGCCTGAACTTGGAGGACCA				
U_157_Antisense_R1	TGCTGTGGCAAGTTTTTCCT				
RGS5		No	113bp	RT-qPCR (Fig. S4B)	
U_157_Sense_F1	CACAGCAGCTTTGCTCAGTG				
U157_Sense_R1	GGAAAGAAGGCCTTCGGACA				
INTS12		No	53bp	RT-qPCR (Fig. S4B)	
29_D_INTS12_F1	GGAGCCACCCAAAATTTCAAGC				
29_D_INTS12_R1	TGGGCTCTTGCTTAATGGAAATG				
NPNT		No	56bp	RT-qPCR (Fig. S4B)	
29_U_NPNT_F1	TGGCAAACTGTCAGTATGGC				
29_U_NPNT_R1	GCACTGGCACCGTATTTGTC				
PES1		No	65 bp	RT-qPCR (Fig. S4B)	
22_D_PES1_F1	CTACCTACTGCCCCCATTCA				
22_D_PES1_R1	AATCACGGGTCCAACTGTGT				
TCN2		No	50 bp	RT-qPCR (Fig. S4B)	
22_U_TCN2_F1	GAGCAGAGAGCCAAGCATCT				
22_U_TCN2_R1	CCAGACTTGGCCAGAAAGAC				

PCR products	Primer sequence	Intron	Expected length	Application	References
CFAP97		No	53bp	RT-qPCR (Fig. S4B)	
31_D_CFAP97_F1	TGCCATCATGGATCAGTTTGG				
31_D_CFAP97_R1	AAGAATGGTCCACTTCACCTTC				
SNX25		No	87bp	RT-qPCR (Fig. S4B)	
31_U_SNX25_F1	GTCAGCTGGATTTTCAGTGAGC				
31_U_SNX25_R1	TGGTGCCAACTTCCCATTTG				
ZFAND1		No	50bp	RT-qPCR (Fig. S4B)	
27_D_ZFAND1_F1	TACCCATGCTCTTTCAAAGACTG				
27_D_ZFAND1_R1	ATAACTGCCACAAGTTCTCTCTC				
CHMP4C		No	134bp	RT-qPCR (Fig. S4B)	
27_U_CHMP4C_F1	AAGCTTGGAATGGGGCAATG				
27_U_CHMP4C_R1	TCATAATGGCCTGGGAGTGTTC				
TIMM9		No	63bp	RT-qPCR (Fig. S4B)	
21_D_TIMM9_F1	TGGCCAACCACGATAGAGAAG				
21_D_TIMM9_R1	GCAGCTGTTGGCAATCTTTC				
KIAA0586		No	65bp	RT-qPCR (Fig. S4B)	
21_U_KIAA0586_F1	AGGATGCTGTTACTCAGGAGAC				
21_U_KIAA0586_R1	AGCCACGGTTGTAGCTGAATC				
RAD51		No	78 bp	RT-qPCR (Fig. S4B)	
AD_012_Sense_F1	GCCAACGATGTGAAGAAATTGG				
AD_012_Sense_R1	TAGCTCCTTCTTTGGCGCATAG				
RAD51-AS1		No	89 bp	RT-qPCR (Fig. S4B)	
AD_012_Antisense_F1	ACCATCGCAGCTGGACTATTAG				
AD_012_Antisense_R1	ACCAGAAGTGCCAAAAGCTG				
EIF3I		No	56bp	RT-qPCR (Fig. S4B)	
26_D_EIF3I_F1	ACCTGCGGTTTTGACTTTGG				
26_D_EIF3I_R1	ATCTGCTTGTCCGTGGAGAAC				
TMEM234		No	69bp	RT-qPCR (Fig. S4B)	
26_U_TMEM234_F1	TGTGCCCATCTGTAACTCTCTG				
26_U_TMEM234_R1	ATATCTTCTCCAAGGGCCTTCC				
HNF1A		No	79bp	RT-qPCR (Fig. S4B)	
30_D_HNF1A_F1	GCCGAGCCATGGTTTCTAAAC				
30_D_HNF1A_R1	TCTTTGCTCAGCCCTGACTC				

PCR products	Primer sequence	Intron	Expected length	Application	References
HNF1A-AS1		No	70bp	RT-qPCR (Fig. S4B)	
30_U_HNF1A-AS1_F2	ACCTGAAACACCTCGCATTG				
30_U_HNF1A-AS1_R2	TGCCTTTGGGAAATGCAGAC				
MCM7		No	86bp	RT-qPCR (Fig. S4B)	
25_D_MCM7_F1	CAGAGCAGCGCTGTGTATCT				
25_D_MCM7_R1	CTGCCAGACATTGAGCTCCT				
AP4M1		No	130bp	RT-qPCR (Fig. S4B)	
25_U_AP4M1_F1	CGCCTATGTCATTCGGATCT				
25_U_AP4M1_R1	CTTCCTGCCCAGATTCAGAG				
AHSA1		No	65bp	RT-qPCR (Fig. S4B)	
20_D_AHSA1_F1	CTATGGCGCACGCTTATTTT				
20_D_AHSA1_R1	TGGACTGAAGTGTCCAGCAG				
VIPAS39		No	53bp	RT-qPCR (Fig. S4B)	
20_U_VIPAS39_F2	CTCTACTGCTGGCCAAGACC				
20_U_VIPAS39_R2	CACTGCTGCATCTGAGCTTC				
UROD		No	84bp	RT-qPCR (Fig. S4B)	
24_D_UROD_F1	GTGAAGCAGATGCTGGATGA				
24_D_UROD_R1	TTCTGGGTCCATGTCAGGAT				
HECTD3		No	53 bp	RT-qPCR (Fig. S4B)	
24_U_HECTD3_F1	TGACTCAGAACCCACATCCA				
24_U_HECTD3_R1	TGGGGAGGTCGTGGTAGTAG				
PMS2P1		No	52bp	RT-qPCR (Fig. S4B)	
32_D_PMS2P1_F1	GCTTAAGGACTATGGAGTGGATC				
32_D_PMS2P1_R1	CCCACATCCATTGCCTGAAAC				
STAG3L5P		No	53bp	RT-qPCR (Fig. S4B)	
32_U_STAG3L5P_F1	TTGAGGACAGCTTCAATCGC				
32_U_STAG3L5P_R1	CAGTGGTCGTTTTGTTGCTTTC				
01_BCAR3_AS_A1		No	115bp	ChIP-qPCR (Fig. 4L)	
01_BCAR3_AS_A_F1	TTTCAGGCATTGCTCAAGGG				
01_BCAR3_AS_A_R1	TCGGCCACGCTTTATTGTTC				
01_BCAR3_AS_B1		No	100bp	ChIP-qPCR (Fig. 4L)	
01_BCAR3_AS_B_F1	TCATGGGACTTTCAGCAAGC				
01_BCAR3_AS_B_R1	CCATGCCTGCTTACAACATGTG				

PCR products	Primer sequence	Intron	Expected length	Application	References
01_BCAR3_AS_C1		No	105bp	ChIP-qPCR (Fig. 4L)	
01_BCAR3_AS_C_F1	TTGTCCCTTCCAGAGCATACAC				
01_BCAR4_AS_C_R1	TTGGTTACACAGGTGCTTCC				
01_BCAR3_AS_D3		No	48bp	ChIP-qPCR (Fig. 4L)	
01_BCAR3_AS_D_F3	ACAGAGTCTTACTCTATCAC				
01_BCAR3_AS_D_R3	GAGATTGTGCTATTGCACTC				
01_BCAR3_AS_E1		No	105bp	ChIP-qPCR (Fig. 4L)	
01_BCAR3_AS_E_F1	TCATACAGCTGAGCCAGGATG				
01_BCAR3_AS_E_R1	GCATGATGCTGCTGCTTACAG				
02_GPX2_A1		No	145bp	ChIP-qPCR (Fig. 4L)	
02_GPX2_A_F1	TCAGTTCCAGGCCATCATGAC				
02_GPX2_A_R1	CTGCAGTGAGCTATGATTGTGC				
02_GPX2_B1		No	63bp	ChIP-qPCR (Fig. 4L)	
02_GPX2_B_F1	AAAATGAAGCGCCCTTTCCG				
02_GPX2_B_R1	CTCTAGCTGGACTTGGTTTTGC				
02_GPX2_C1		No	80bp	ChIP-qPCR (Fig. 4L)	
02_GPX2_C_F1	AATGGAGCTTTGAGCATCCC				
02_GPX2_C_R1	GCAACCTCCTTGTTCAAACAGC				
02_GPX2_D2		No	68bp	ChIP-qPCR (Fig. 4L)	
02_GPX2_D_F2	CATCCATTGGGAACTGTCTC				
02_GPX2_D_R2	ATTCTCAAGACCAGCTGCTC				
02_GPX2_E1		No	88bp	ChIP-qPCR (Fig. 4L)	
02_GPX2_E_F1	AGAGATGCCAAAGCTTTGCC				
02_GPX2_E_R1	TTGCCTAAGGGTCCGAAGTAAC				

References

1. Weissbein, U., Schachter, M., Egli, D. & Benvenisty, N. Analysis of chromosomal aberrations and recombination by allelic bias in RNA-Seq. *Nat. Commun.* **7,** 12144 (2016).

2. Sagi, I. *et al.* Distinct imprinting signatures and biased differentiation of human androgenetic and parthenogenetic embryonic stem cells. *Cell Stem Cell* **25,** 419–432.e9 (2019).

3. Sakai, S. *et al.* Long noncoding RNA *ELIT-1* acts as a Smad3 cofactor to facilitate TGFβ/Smad signaling and promote epithelial-mesenchymal transition. *Cancer Res.* **79,** 2821–2838 (2019).
